# Human cord blood‐derived regulatory T‐cell therapy modulates the central and peripheral immune response after traumatic brain injury

**DOI:** 10.1002/sctm.19-0444

**Published:** 2020-05-07

**Authors:** Henry W. Caplan, Karthik S. Prabhakara, Akshita Kumar, Naama E. Toledano‐Furman, Cecilia Martin, Louis Carrillo, Nicolas F. Moreno, Andrea S. Bordt, Scott D. Olson, Charles S. Cox

**Affiliations:** ^1^ Department of Pediatric Surgery, McGovern Medical School University of Texas Health Science Center at Houston Houston Texas USA

**Keywords:** cell therapy, central nervous system trauma, cord blood, microglia, neuroinflammation, regenerative medicine, traumatic brain injury, Treg, t‐SNE

## Abstract

Traumatic brain injury (TBI) causes a profound inflammatory response within the central nervous system and peripheral immune system, which contributes to secondary brain injury and further morbidity and mortality. Preclinical investigations have demonstrated that treatments that downregulate microglia activation and polarize them toward a reparative/anti‐inflammatory phenotype have improved outcomes in preclinical models. However, no therapy to date has translated into proven benefits in human patients. Regulatory T cells (Treg) have been shown to downregulate pathologic immune responses of the innate and adaptive immune system across a variety of pathologies. Furthermore, cellular therapy has been shown to augment host Treg responses in preclinical models; yet, studies investigating the use of Treg as a therapeutic for TBI are lacking. In a rodent TBI model, we demonstrate that human umbilical cord blood Treg modulate the central and peripheral immune response after injury in vitro and in vivo.

## Graphical abstract

A rat model of traumatic brain injury was used to determine whether regulatory T cells (Treg) could be used to treat neuroinflammation associated with severe central nervous system trauma. We utilized multicolor flow cytometry 30d after injury to rapidly assess the changes in the phenotype of microglia, which were visualized using a dimensional reduction algorithm (t‐distributed stochastic neighbor embedding). This method clearly maps populations that change as a result of injury but respond to Treg treatment. Further analysis demonstrates that these particular populations express microglia markers that change in expression levels in response to injury, including CD11bc (pictured here). This response is ameliorated by Treg treatment 24 hours after injury.

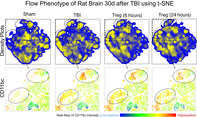

**Significance statement**

This study demonstrates that human regulatory T cells (Treg) expanded from umbilical cord blood can reduce neuroinflammation associated with traumatic brain injury. A single dose of Treg can cause long‐term changes in brain microgliosis in chronic TBI.

## 1 INTRODUCTION

Traumatic brain injury (TBI) is a leading cause of morbidity and mortality.^1^, ^2^ TBI causes a profound inflammatory response within the central nervous system (CNS) and peripheral immune system, which contributes to secondary brain injury and further morbidity and mortality.^3^, ^4^ Microglia, the resident myeloid cells in CNS, are key mediators of the inflammatory response within the brain after TBI.^3^, ^5^ Previously, we have demonstrated that cell therapy including mesenchymal stem cells (MSC) and multipotent adult progenitor cells (MAPC) downregulate microglia activation and polarize them toward a reparative/anti‐inflammatory phenotype and have improved outcomes in preclinical models.^6^, ^7^, ^8^ The mechanism of action of cell therapy likely involves interaction with and modulation of the endogenous immune system, specifically regulatory T cells (Treg).^9^ Treg maintain self‐tolerance and regulate immune responses, and Treg dysfunction is involved in many immunologic‐related pathologies from graft vs host disease to multiple sclerosis.^10^, ^11^ Several clinical trials using Treg therapy are now under way in several disease processes. However, investigations into the use of Treg therapy for TBI are lacking. Here, we evaluated the ability of human Treg to modulate the immune response to TBI both in vitro and in vivo. Based on previous work with progenitor cells, such as MSC and MAPC, we evaluated to the ability of human umbilical cord blood (UCB)‐derived Treg to attenuate proinflammatory cytokine production by stimulated human and rodent immune cells, as this has been predicative of successful translation of cell therapies into animal models.^7^, ^9^, ^12^, ^13^, ^14^ Furthermore, in a rodent TBI model, we investigated the effects UCB Treg therapy on peripheral immune cell populations, blood brain barrier (BBB) permeability, and microglia activation in vivo. We hypothesized that Treg infusion would modulate the central and peripheral immune response after injury, improve BBB permeability, and attenuate microglia activation.

## 2 MATERIALS AND METHODS

All protocols involving the use of animals were in compliance with the National Institutes of Health Guide for the Care and Use of Laboratory Animals and were approved by the University of Texas Health Science Center Institutional Animal Care and Use Committee (AWC‐18‐0121). Human peripheral blood was obtained after informed consent from healthy human adult donors according to a protocol approved by the Institutional Review Board (IRB) (HSC‐MS‐10‐0190). Human UCB was obtained via a Material Transfer Agreement (MTA) with the MD Anderson Cord Blood Bank.

### 2.1 Animals

7Male Sprague Dawley Rats (225‐250 g, Envigo Labs, Indianapolis, Indiana) were the source of CNS and splenic tissue. The usage of the animals was approved by the Animal Welfare Committee at University of Texas Health Science Center at Houston, Texas, protocol: AWC‐18‐0121. Animals were handled in accordance with the standards of the American Association for the Accreditation of Laboratory Animal Care. Five‐week‐old rats were housed in pairs under 12‐hour light/dark cycles in temperature‐controlled conditions. Water and standard rodent laboratory chow were accessible ad libitum.

### 2.2 Treg isolation, expansion, and characterization

#### 2.2.1 *Human UCB mononuclear cell isolation*

Two individual donor UCB units were used and kept separate throughout the isolation and expansion process. Mononuclear cells (MNC) were isolated from the human UCB using 50‐mL SepMate‐50 peripheral blood MNC (PBMC) isolation tubes (STEMCELL Technologies, Vancouver, Canada) according to their modified and simplified protocol adapted from conventional Ficoll isolation. Briefly, 15 mL of Ficoll‐Paque density gradient medium (GE Healthcare, Chicago, Illinois) was added to each tube. Human UCB was mixed 1:1 with phosphate buffered saline (PBS; Lonza, Basel, Switzerland), and 30 mL was carefully pipetted on top of the Ficoll‐Paque. The tubes were centrifuged for 10 minutes at 1200*g* with the brake on. The top layer was quickly poured off into another 50‐mL centrifuge tube. The cells were washed with PBS and centrifuged at 400*g* for 8 minutes with the brake on. The cells were then counted and viability was assessed using the NucleoCounter NC‐200 and Via2‐Cassettes (Chemometec, Allerod, Denmark). A repeat wash was performed, and the cells were suspended in buffer consisting of PBS, 2 mM ethylenediaminetetraacetic acid (EDTA), and 0.5% human serum albumin (HSA; Baxter, Deerfield, Illinois).

#### 2.2.2 *Treg isolation*

Isolation of Treg was performed using a human Treg isolation kit as per the manufacturer instructions (Miltenyi Biotec, Auburn, California, 130‐091‐301). Briefly, non‐CD4+ cells were depleted from the resultant MNC by incubating the cells with the CD4+ T‐cell Biotin Antibody Cocktail (containing biotin‐conjugated monoclonal anti‐human antibodies against CD8, CD14, CD15, CD16, CD19, CD36, CD56, CD123, TCRγ/δ, and CD235a) and Anti‐Biotin MicroBeads, followed by passage of cells through an LD column (Miltenyi Biotec). The non‐CD4+ cells remained within the column, while the effluent, consisting of the unlabeled CD4+ cells, was collected and counted. The cells were then washed, centrifuged at 300*g* for 5 minutes, and resuspended in buffer. CD25+ cells were positively selected by incubating the cell suspension with CD25 MicroBeads. The cell suspension was then passed through an MS column; positive cells remained in the column and were subsequently flushed out of the column. This process was repeated to improve purity. Cells were counted, and viability was assessed.

#### 2.2.3 *Treg expansion*

The positively selected CD4+CD25+ cells were resuspended in Treg expansion media at an initial seeding density of 1 × 10^6^ cells/mL. Treg expansion media consisted of HyClone RPMI 1640 (GE Healthcare), 5% human AB serum (Thermo Fisher Scientific, Waltham, Massachusetts), 1% GlutaMAX (Gibco, Thermo Fisher Scientific), 10 μg/mL of gentamicin, 100 nM rapamycin (Thermo Fisher Scientific), and 500 IU/mL interleukin (IL)‐2 (CellGenix, Freiburg, Germany). On day 0 (day of isolation), 100 μL of cell suspension was added to each well of a round bottom 96‐well plate. CD3/CD28 MACSiBead Particles were prepared by washing 200 μL of bead suspension with Treg expansion media and centrifuging at 300*g* for 5 minutes. Next, 200 μL of Treg expansion media was added to the bead pellet and resuspended. Then, 20 μL of bead suspension (ratio of 4:1 bead: cell) was added to each well.

On day 1, 100 μL of Treg expansion media was added to each well. Cells were split every 2 to 3 days to maintain a cell density of 5 × 10^5^ cells/mL and transferred to increasing sized culture vessels based on cell count. On day 15, the cells were restimulated with CD3/CD28 beads at a ratio of 4:1 bead: cell. On day 21, the cells were counted and washed with PBS. The cells were centrifuged at 300*g* for 5 minutes, resuspended in buffer, and transferred to a 15‐mL conical tube. The tube was placed in a MACSiMAG magnet Separator to remove the CD3/28 beads (Miltenyi Biotec). The beads were allowed to adhere to the wall of the tube, and the supernatant was removed and placed into a new tube. The cells were centrifuged again at 300*g* and resuspended in cryopreservation media (10% HSA and 90% dimethyl sulfoxide (DMSO); Thermo Fisher Scientific) at a cell density of 5 × 10^6^ cells/mL. Then, 1 mL of cell suspension was placed in 1.5‐mL cryovials (CORNING, Corning, New York). The cryovials were placed in a Mr Frosty Freezing Container (Nalgene Nunc, Rochester, New York) and stored at −80°C for 24 hours. They were then transferred to liquid nitrogen vapor phase storage and stored until further use.

#### 2.2.4 *Treg thaw procedure*

Prior to use, Treg were freshly thawed and washed with PBS. For in vitro experiments, the cells were centrifuged at 300*g* for 5 minutes, resuspended in 1 mL of the assay‐specific media. Cell counts and viability were obtained with the NuceloCounter. For in vivo experiments, the cells were centrifuged at 300*g* for 5 minutes, resuspended in PBS, placed on ice prior to infusion. Cell counts and viability were obtained with the NucleoCounter.

#### 2.2.5 *Treg flow cytometry immunophenotyping*

Flow cytometry was performed on day 0, day 15, and on day of infusion to evaluate the cell population and identify Treg (Figure S1). The multicolor panel was composed of CD3 (FITC), CD4 (PE), CD8 (APC), CD25 (PE‐Cy7), CD127 (APC‐R700), CD19 (BV510), and 7‐AAD cell viability solution. Data were acquired with a Gallios Flow Cytometer (Beckman Coulter, Brea, California) and analyzed with Kaluza software version 2.1 (Beckman Coulter). Cell debris and doublets were excluded using forward scatter (FSC) and side scatter (FSC) parameters, and dead cells were excluded on the basis of 7‐aminoactinomycin D (7‐AAD) staining. Treg cells were identified as CD3+CD4+CD25+CD127_dim_ cells.

### 2.3 In vitro experiments

#### 2.3.1 *Human PBMC isolation and activation assay*

Fresh human peripheral blood was collected under university‐approved IRB protocol (MSC‐10‐0190). PBMCs were isolated using the same protocol as described above, using SepMate‐50 tubes and Ficoll‐Plaque. The PBMCs were then washed with PBS, counted, and suspended in RPMI with 10% fetal bovine serum (FBS). To evaluate the suppressive ability of Treg, we performed a PBMC activation assay, in which 2 × 10^5^ PBMCs were added to a 96‐well plate and activated using CD3/28 biotinylated beads (Miltenyi Biotec) following the manufacture's protocol. Treg were added at various Treg:PBMC ratios. The culture supernatant was collected at 72 hours. The samples were analyzed using human enzyme‐linked immunosorbent assay (ELISA) kits for interferon gamma (IFN‐γ) (Biolegend, San Diego, California) per the manufacturer's protocol.

#### 2.3.2 *Rat splenocyte isolation and activation assay*

Splenocytes were isolation using a modified version of previously described protocols.^12^, ^13^ Briefly, a spleen was harvested from a naïve rat and a rat 24 hours postcontrolled cortical impact (CCI; described in full detail below) under anesthesia. The spleen was washed in 10 mL of PBS, homogenized using a gentleMACS Dissociator (Miltenyi Biotech). The cells were filtered through a 70‐μm filter and centrifuged at 400*g* for 5 minutes. The cells were washed once again in PBS and resuspended in RPMI with 10% FBS. To evaluate the suppressive ability of Treg, splenocytes (2 × 10^6^ cells/mL) were activated with lipopolysaccharide (LPS) or concanavalin A (Con A), alone, or in the presence of Treg at a 1:2 Treg:splenocyte ratio in 96‐well culture plates. The culture supernatant was collected 24 hours after LPS activation or 72 hours after Con A activation. The samples were analyzed utilizing rat specific tumor necrosis factor alpha (TNF‐α) and IFN‐γ ELISA kit (BD Biosciences, San Jose, California) per the manufacturer's protocol.

#### 2.3.3 *Rat microglia isolation and activation assay*

A naïve rat brain was harvested and processed as previously described by our lab utilizing the Neural Tissue Dissociation Kit and GentleMACS Dissociator (Miltenyi Biotec).^15^ Myelin was removed by Percoll centrifugation. The brain cells were counted and 2.5 × 10^5^ cells were plated in a 24‐well plate coated with cell matrix basement membrane gel (ATCC, Manassas, Virginia) in microglia media (Dulbecco's modified eagle medium + 10% FBS). The cells were allowed to rest for 6 days, with media changes every 2 days, prior to the experiment.

On day 6, microglia cells in culture were then stimulated with LPS (1 μg/mL). The same splenocytes from the naïve and post‐CCI rats as described above were used in this experiment. 2 hours after stimulation, Treg (1:2), naïve splenocytes (Naïve Sp; 1:2), or splenocytes 24 hours post‐CCI (CCI Sp; 1:2) were added to the wells. Treg + Naïve Sp and Treg + CCI Sp were added to additional wells. Naïve microglia received neither LPS nor cells. Culture supernatant was collected at 18 hours after LPS stimulation. ELISA was performed for rat TNF‐α.

In addition, immunohistochemistry (IHC) was performed to identify microglia cells. Briefly, in the well plate, cells were rinsed with PBS and fixed with 4%. The wells were washed twice in PBS with 0.01% Triton X‐100 (PBST; T‐8787; Sigma‐Aldrich, St. Louis, Missouri, http://www.sigmaaldrich.com) for 5 minutes and blocked for 1 hour at room temperature (RT) in 3% goat serum (Jackson Immunoresearch Laboratories, West Grove, Pennsylvania) in PBST. IBA1, a rabbit polyclonal primary antibody, was used to identify microglia (1:200; Wako Chemicals USA, Richmond, Virginia). The antibody was prepared in PBTB (PBS with 0.01% Triton X‐100, 2% bovine serum albumin; Sigma‐Aldrich) and 1% goat serum. The wells were incubated overnight at 4°C. The next day, the wells were rinsed briefly and then washed with PBST, and incubated with a goat anti‐rabbit IgG secondary antibody (1:200; red/568; Molecular Probes, Invitrogen, Waltham, Massachusetts). The wells were incubated with Hoechst dye (Molecular Probes), diluted 1:1000 in PBST, for 10 minutes and then rinsed four times with PBST. Imaging was performed on a Nikon fluorescent microscope (TE2000‐U) and NIS‐Elements imaging software (Nikon, Tokyo, Japan).

### 2.4 In vivo experiments

#### 2.4.1 *Experimental groups*

**FIGURE 1:**
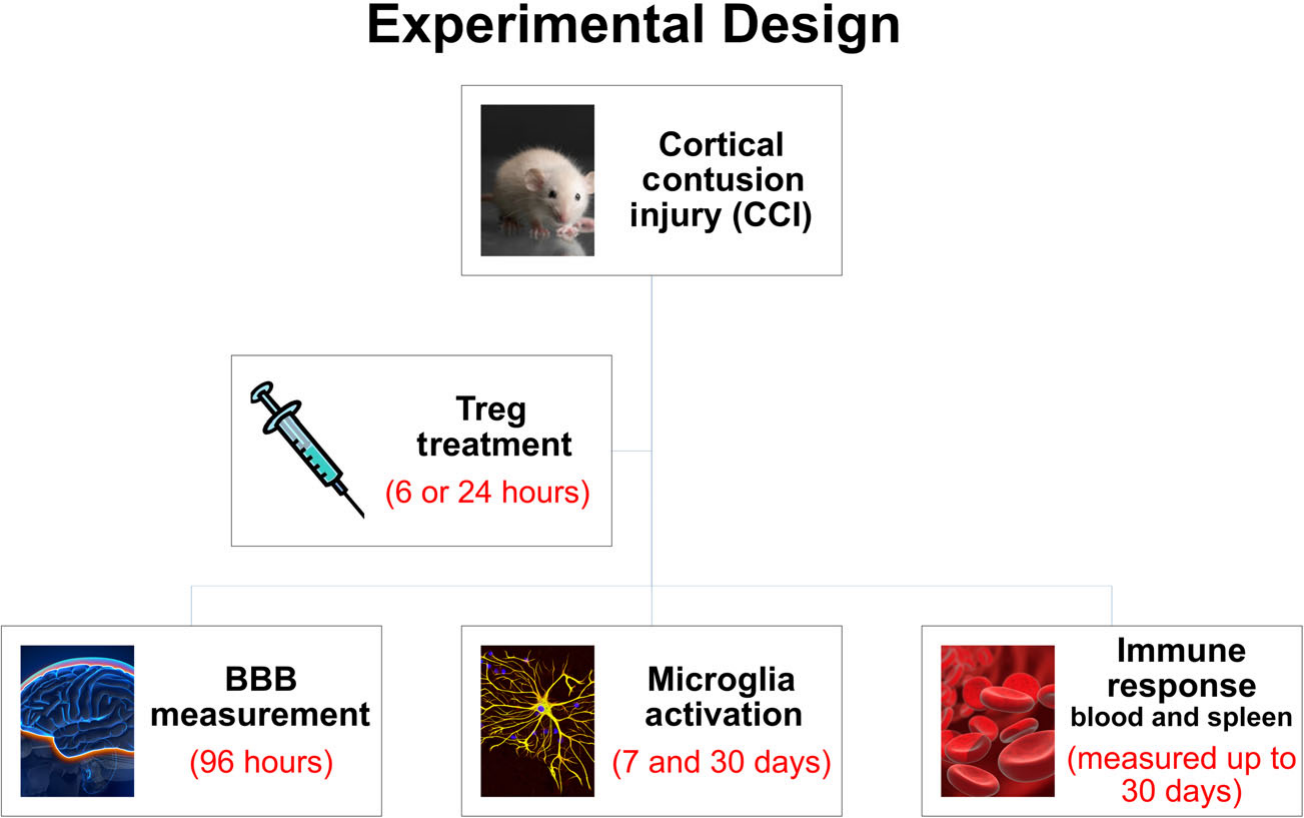
Experimental design and timing of the performed in vivo experiments

Experiments were conducted in multiple cohorts at three different time points based on the outcome measurement (Figure 1). Experiments lasted either 96 hours to assess BBB permeability, 7 days to assess subacute microglia activation, or 30 days to assess chronic microglia activation.

#### 2.4.2 *CCI model*

Animals were anesthetized with 4% isoflurane and oxygen in a vented chamber and then maintained at 2% to 3% isoflurane for the duration of the procedure. The animal was then secured on a stereotactic frame and the surgical site was prepped with alcohol and iodine solution. Subcutaneous 0.25% bupivacaine was administered prior to incision for local anesthesia. A midline cranial incision was made, and the right‐sided musculature and soft tissue were bluntly dissected away for exposure of the calvarium. A 7‐mm diameter craniectomy was performed between the right coronal and lambdoid sutures. A CCI device (Impact One Stereotaxic Impactor, Leica Microsystems, Buffalo Grove, Illinois) was utilized to administer a standardized and unilateral severe brain injury as previously described.^16^, ^17^ Severe injury parameters included a depth of 3.1 mm, impact velocity of 5.6 m/s, and a dwell time of 150 microseconds using a 6‐mm diameter impactor tip to the parietal association cortex. Immediately after the injury, the incision was closed using sterile wound clips and animals were allowed to recover in newly cleaned microisolator cages provided by the University Center for Laboratory Medicine and Care (CLAMC). Sham injuries were performed by anesthetizing the animals, making the midline incision, and separating the skin, connective tissue and aponeurosis from the cranium. The incision was closed using sterile wound clips.

#### 2.4.3 *Treg infusion*

Treg were thawed and washed as described above. Treg were prepared so that each animal in the treatment group received a single dose of 10 × 10^6^ cells/kg in 1 mL of sterile PBS. All treatment animals underwent CCI and then received Treg infusion, via tail vein, at either 6 hours (Treg 6 hours) or 24 hours (Treg 24 hours) after injury.

#### 2.4.4 *BBB permeability measurement*

BBB permeability was assed as previously described.^17^, ^18^ Briefly, 1 mg/kg Alexa 680 (Life Technologies, Thermo Fisher Scientific) was injected via tail vein 30 minutes before animals were euthanized at 96 hours after CCI. Following exsanguination, PBS and 4% paraformaldehyde perfusion, the brains were harvested. The brains were then sliced in 2‐mm coronal sections and scanned for Alexa 680 using a LiCor Odyssey infrared laser scanner (LI‐COR, Lincoln, Nebraska). Quantitative measurements of dye extravasation were determined by thresholding to remove background followed by measuring mean intensity of the slices and normalizing to the sham‐injured animals (ImageJ).

#### 2.4.5 *Blood and splenocyte flow cytometry immunophenotyping*

Flow cytometry for blood and spleen was performed to characterize lymphoid and myeloid cell populations. The antibody panel consisted of the following cell surface markers: anti‐CD3‐FITC, anti‐CD25‐PE, anti‐CD8a‐PerCP, anti‐CD11bc‐PECy7, anti‐RT1B‐APC, anti‐CD4‐APCCy7, anti‐CD45RA‐V450. Myeloid cells were identified as CD11bc‐positive, CD3‐negative, CD45RA‐negative cells. B cells were identified as CD45RA‐positive, CD3‐negative, CD11bc‐negative cells. T cells were identified as CD3‐positive, CD11bc‐negative, CD45RA‐negative cells. Further T‐cell subsets were identified using the CD4, CD8, and CD25 markers. Blood was collected via tail vein, under anesthesia at various time points after injury, or direct cardiac puncture during euthanasia in heparinized collection vials. Next, 100 μL of blood was added directly to flow tubes and stained with the antibody panel. Spleens were harvested from animals at the time of euthanasia and immediately weighed. Splenocytes were isolated as described above and stained with the antibody cocktail. In addition, a control sample of the human Treg was also analyzed with the rat flow cytometry panel to ensure that the human cells did not stain positive on our rat antibody panel (Figure S4). Data for the blood and splenocyte samples were acquired on a Galios Flow Cytometer (Beckman Coulter). Subsequent data analyses were completed utilizing Kaluza software (Beckman Coulter). Comparisons between means of each group were analyzed using ordinary one‐way analysis of variance (ANOVA) with Sidak's multiple comparisons test. Robust regression and outlier removal (ROUT) analyses identified outliers in the data, which were excluded in the final analysis. All group data are presented as mean ± SE. Values of *P* ≤ .05 were considered significant. Statistical significance is indicated with *P ≤ .05, **P ≤ .01, ***P ≤ .001, and ****P ≤ .0001.

#### 2.4.6 *Microglia flow cytometry immunophenotyping*

Animals were euthanized at either 7 or 30 days after injury. Upon sacrifice, brains were harvested and processed as described above.

The isolated brain cells were immunophenotyped using a modified version of our previously published microglia immunophenotyping strategy using the following cell surface antibodies: anti‐CD45, anti‐CD11bc, anti‐P2Y12, anti‐CD32, anti‐RT1B, and anti‐CD163.^19^ Briefly, the cells were stained with the Ghost live/dead reagent and incubated for 15 minutes at RT in the dark. The cells were then washed with 1 mL of staining buffer and centrifuged at 400*g* for 5 minutes. The supernatant was removed, and the cells were then stained with the antibody mix mentioned in Table 1 and incubated for 30 minutes at RT in the dark. Cells were washed again with staining buffer and centrifuged at 400*g* for 5 minutes. The supernatant was removed, and the secondary antibody mix was added. The cells were incubated for 20 minutes at RT in the dark. The cells were then gently vortexed, and 25 μL of counting control beads (Cyto‐Cal) were added. Data were acquired with LSR‐II flow cytometer with Diva acquisition software (BD Biosciences). Fluorescence spillover compensation values were generated using VersaComp Antibody Capture Beads (Beckman Coulter).

**TABLE 1:** Multicolor flow cytometry microglia/myeloid cell panel

Fluorochrome	Antibody	Clone	Vendor	Catalog #	Purpose
APC‐Cy7	CD45	OX‐1	BD	561586	Leukocyte common antigen
					Microglia (+), macrophage (+)
PE‐Cy7	CD11b/c	OX‐42	BD	562222	General myeloid cell marker
					Microglia (+), macrophage (+)
BV421	P2Y12	n/a	Alomone Labs	APR‐020‐F	Mediates microglia chemotaxis
					Microglia (+), macrophage (−)
PE	CD32	D34‐485	BD	562189	Associated with phagocytosis, activates inhibitory signaling
					Microglia (+), macrophage (+)
Alexa Fluor 647	RT1B	OX‐6	BD	562223	Major histocompatibility complex class II marker, antigen presentation
					Microglia (+), macrophage (+)
PerCP Cy5.5	CD163	ED2	Bio Rad	MCA342R	Associated with hemoglobin clearance Microglia (+), macrophage (+)
BV510	Ghost	n/a	Tonbo	13‐0870‐T100	Live/dead reagent

### 2.5 Data analysis

#### 2.5.1 *Microglia/macrophage cell gating strategy*

Traditional flow cytometry analysis was also performed with FlowJo vr10.6.1. In order to identify P2Y12+ microglia, live cells were initially gated by P2Y12 expression and then gated on CD11bc and CD45 (Figure S3). CD11‐positive cells were then gated on phenotypic markers CD32, RT1B, and CD163; the percentage of parent populations and median fluorescent intensity (MFI) were calculated. In addition, in order to ensure identification of all microglia and myeloid cells, live cells were gated on CD11bc and CD45 without P2Y12. CD11bc‐positive cells were then gated on phenotypic markers CD32, RT1B, and CD163; the percentage of parent populations and MFI were calculated.

#### 2.5.2 *t‐Distributed stochastic neighbor embedding analysis*

t‐Distributed stochastic neighbor embedding (t‐SNE) analysis was performed with FlowJo vr10.6.1 (FlowJo, LLC, Ashland, Oregon). Briefly, live cells were gated on all samples. Within each sample, live cell events were randomly downsampled to 3000 events, and analysis was run on equal numbers of events per sample. The individual sample files were concatenated to link them together into a single‐standard file. t‐SNE was run using the FlowJo plugin, which included all fluorophores listed in Table 1 as parameters. Unique t‐SNE was created for each time point, 7 and 30 days post‐CCI. In each t‐SNE, all samples and groups were derived from the same t‐SNE run.

#### 2.5.3 *Statistical analysis: In vivo microglia data*

Microglia flow cytometry data were analyzed with GraphPad Prism software version 8.2 (GraphPad Software, Inc., La Jolla, California). Absolute cell counts per hemisphere were calculated using the reference formula adjusted for volume of beads used per samples and brain hemisphere weight:

$$\text{Absolute cell}/\mathrm{mg}=\left(\text{Cell count}/\text{bead count}\right)\times \left(25\;000/\text{brain weight}\right).$$

Comparisons between means of individual hemispheres of each group were analyzed using ordinary one‐way ANOVA with Sidak's multiple comparisons test. ROUT analyses identified outliers in the data, which were excluded in the final analysis. All group data are presented as mean ± SE. Values of *P* ≤ .05 were considered significant. Statistical significance between sham and CCI is indicated with #*P* ≤ .05, ##*P* ≤ .01, ###*P* ≤ .001, and ####*P* ≤ .0001. Statistical significance between CCI and Treg 24 hours is indicated with *P ≤ .05, **P ≤ .01, ***P ≤ .001, and ****P ≤ .0001. Replicates: 7d cohort, N = 8; 30d cohort, N = 6.

#### 2.5.4 *Statistical analysis: In vitro data, in vivo blood and spleen data*

ELISA and flow cytometry data were analyzed with GraphPad Prism software version 8.2 (GraphPad Software, Inc.). Comparisons between means groups were analyzed using ordinary one‐way ANOVA with Tukey's multiple comparisons test. All group data are presented as mean ± SE. Values of *P* ≤ .05 were considered significant. Statistical significance is indicated with *P ≤ .05, **P ≤ .01, ***P ≤ .001, and ****P ≤ .0001. All in vitro measurements were performed using biological triplicate cultures.

## 3 RESULTS

### 3.1 Immunomodulatory effects of Treg in vitro

**FIGURE 2:**
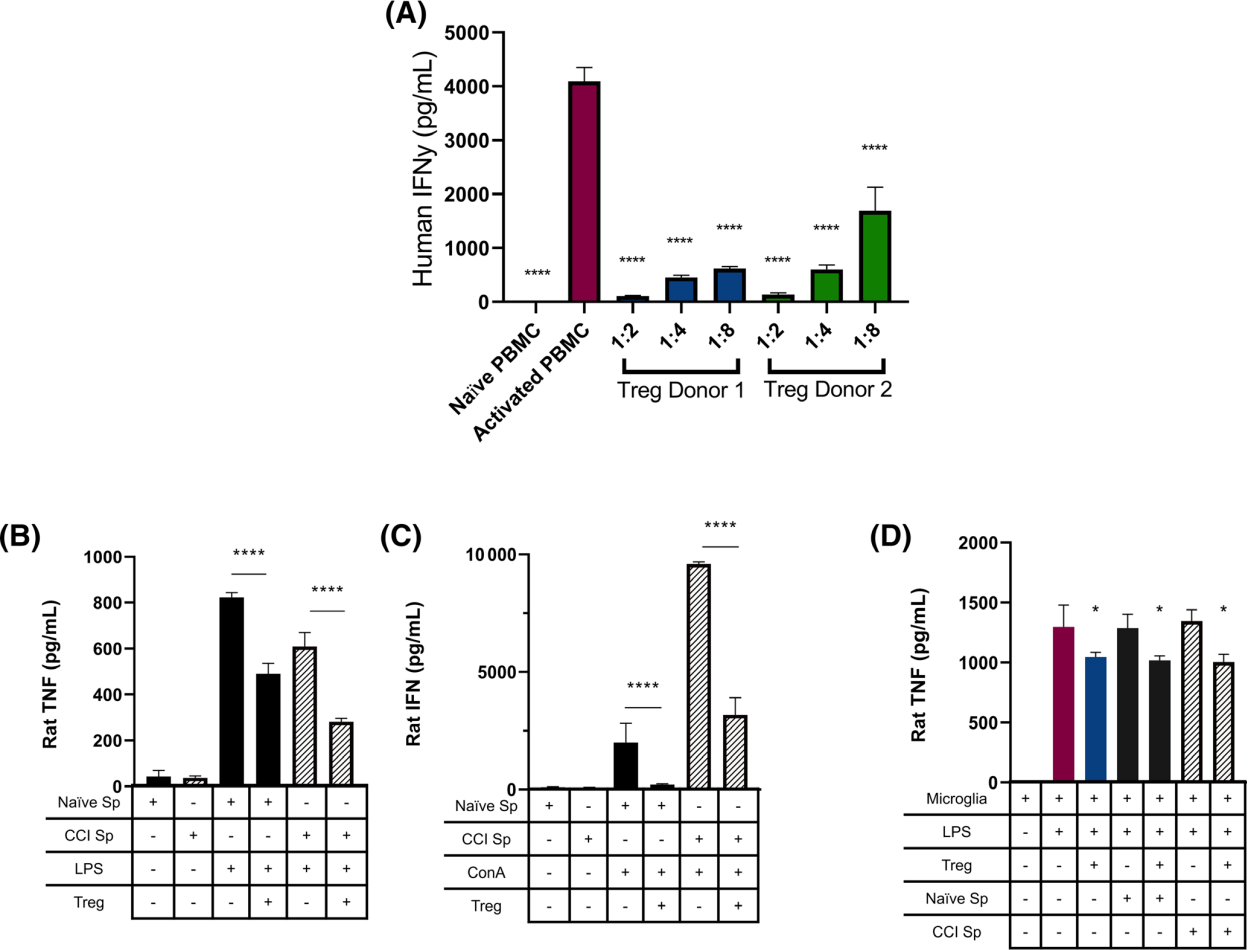
Characterization of the suppressive potential of human Treg on human and rat cells in vitro. A, IFN‐γ production (ELISA) by human PBMC after CD3/CD28 bead. B, TNF‐α production (ELISA) by naïve and post‐CCI rat splenocytes after LPS stimulation. Naïve splenocytes demonstrated a greater response compared with post‐CCI splenocytes. Treg (1:2 Treg:splenocyte ratio) significantly reduced TNF‐α production by both naïve and post‐CCI splenocytes. C, IFN‐γ production (ELISA) by naïve and post‐CCI rat splenocytes after Con A stimulation. Post‐CCI splenocytes demonstrated a greater response compared with naïve splenocytes. Treg (1:2 Treg:splenocyte ratio) significantly reduced IFN‐γ production by both naïve and post‐CCI splenocytes. D, Effect of Treg and rat splenocytes on rat microglia TNF‐α production (ELISA) after LPS stimulation. All samples run in triplicate. Statistical significance is indicated with *P ≤ .05, **P ≤ .01, ***P ≤ .001, and ****P ≤ .0001. CCI, controlled cortical impact; Con A, concanavalin A; IFN‐γ, interferon gamma; LPS, lipopolysaccharide; PBMC, peripheral blood mononuclear cells; TNF‐α, tumor necrosis factor alpha; Treg, regulatory T cells

To demonstrate their anti‐inflammatory potential, we first studied the effect of human UCB‐derived Treg, from two different donors, on inhibition of proinflammatory cytokine from activated human PBMCs. Coculture of Treg with CD3/CD28‐activated PBMC showed a statistically significant reduction in IFN‐γ production compared with the activated control (Figure 2A). Higher ratios of Treg:PBMC result in stronger suppression of IFN‐γ production across both donors, demonstrating a direct dose‐response.

Given the critical role we believe the spleen and splenocytes play in regulating the immune response after TBI, we further examined the effect of Treg on immune responses of rat splenocytes, both naïve (Naïve Sp) and 24 hours post‐CCI (CCI Sp), in order to better understand the role of peripheral immune cells after TBI. Specifically, we used LPS to activate the innate immune response and Con A to activated the adaptive immune response.^20^ Both Naïve Sp and CCI Sp demonstrated the ability to produce an innate and adaptive immune response, as measured by TNF‐α and IFN‐γ, respectively (Figure 2B,C). Interestingly, after CCI, the splenocytes demonstrate an increase in the adaptive response and a corresponding decrease in the innate response. Therefore, at 24 hours after CCI, the adaptive immune response is primed and increasingly active. Treg (donor 1) were used at a 1:2 Treg:splenocyte ratio. Coculture with Treg significantly reduced both TNF‐α production after LPS activation and IFN‐γ production after Con A activation (Figure 2B,C).

Finally, to assess whether Treg can directly attenuate microglia proinflammatory cytokine production, we cocultured Treg with rat microglia after LPS stimulation. Furthermore, we cocultured rat microglia with Naïve Sp and CCI Sp, with and without Treg, in order to examine the effects of splenocytes on activated microglia. Immunohistochemical staining demonstrated that the vast majority of cultured brain cells, prior to addition of Treg or splenocytes, were Iba1+ microglia (Figure S2). Treg significantly suppressed TNF‐α production by activated microglia (Figure 2D). Neither naïve Sp nor CCI Sp coculture with microglia had an effect on TNF‐α production. However, coculture of Treg + Naïve Sp and Treg+CCI Sp with microglia effectively attenuated TNF‐α production.

### 3.2 t‐SNE analyses demonstrate that Treg alter the microglia populations at 30 days, but not 7 days post‐CCI

t‐SNE is a dimension reduction technique that allows for visualization of high‐dimensional data and has become an increasingly popular tool in analysis of high‐dimension mass cytometry and flow cytometry data.^21^, ^22^ With the respect to flow cytometry, t‐SNE produces a two‐dimensional map of the high‐dimension data, and the proximity of one cell to another reflects their similarity in high‐dimensional space.^23^ Therefore, distinct populations of cells will appear as distinct clusters. Subsequent analysis of clusters allows for effective identification of cell types and subpopulations. Here, we have utilized t‐SNE to identify changes in cell populations over time after injury and after Treg infusion.

**FIGURE 3:**
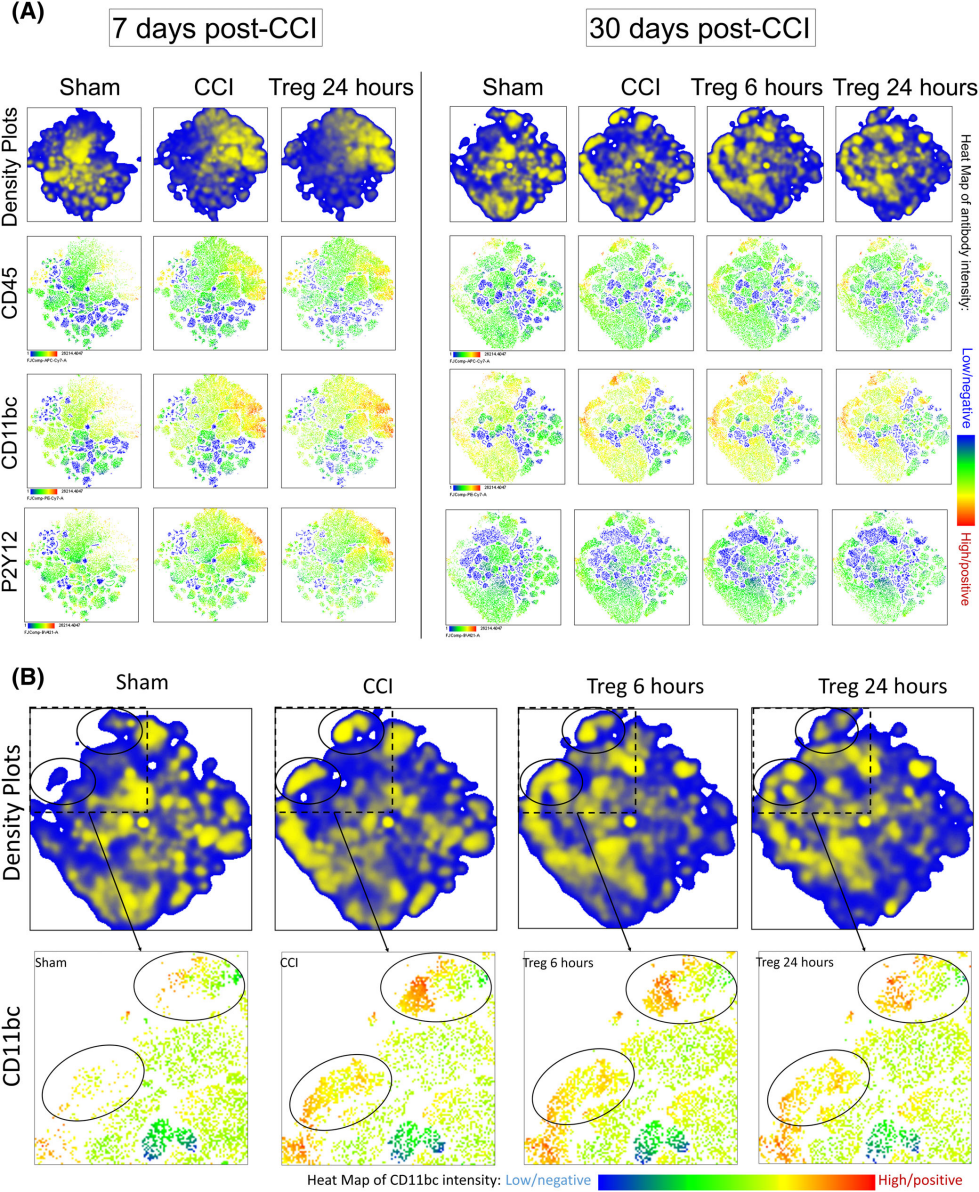
t‐SNE visualization of changes in microglia populations after CCI and treatment. A, Visualization of changes in cell clusters at 7 days post‐CCI (left) and at 30 days post‐CCI (right). At 7 days post‐CCI, there are clear differences between the sham and CCI plot density plots (top row); however, there are no clear differences between the CCI and Treg 24 hours plots. The antibody heat maps demonstrate that these CCI‐specific cell clusters are positive for CD45, CD11bc, and P2Y12, indicating that these cells are microglia. At 30 days post‐CCI, there are persistent visual differences between the sham and CCI density plots; furthermore, the antibody heat maps demonstrate that these cell clusters are also microglia. B, Detailed visualized of CCI‐specific cell clusters (black circles) at 30 days post‐CCI (top). Below, these cell clusters are visualized in more detail with the CD11bc antibody heat map. Within these clusters, there are clear differences between the sham and CCI plots, indicating that the CCI results in long term changes in microglia populations at 30 days after injury. Furthermore, there are clear differences between the CCI and Treg 24 hours groups, with fewer cells present in the treatment group. Replicates: 7d cohort, N = 8; 30d cohort, N = 6. CCI, controlled cortical impact; Treg, regulatory T cells; t‐SNE, t‐distributed stochastic neighbor embedding

At 7 days post‐CCI, there are clear visual changes in the ipsilateral hemisphere after injury, as evidenced by changes in density plots and the heat maps of the microglia phenotypic markers (Figure 3A). However, there are no visual changes between CCI and Treg 24 hours. At 30 days post‐CCI, there are persistent visual changes in the ipsilateral hemisphere after injury as demonstrated by the density plots, with new cell clusters present after injury (Figure 3A). Furthermore, there are differences in these CCI‐specific cell clusters between the CCI and Treg 24 hours groups (Figure 3B). Subsequent analysis revealed that these highlighted cell clusters are microglia, as they are positive for P2Y12, CD45, and CD11bc. Examination of the CD11bc antibody heat map, which demonstrates expression intensity, of the CCI‐specific cell clusters reveals that there are fewer cells present in the Treg 24 hours compared with CCI (Figure 3B).

While t‐SNE relies on visual interpretation of the data, it does provide an unbiased method of mapping changes in cell populations after injury and Treg infusion. Furthermore, these findings corroborate our data from traditional flow cytometry analysis in an unbiased manner.

### 3.3 Treg decreases chronic microgliosis and has mixed effects on microglia phenotypic markers at 30 days post‐CCI, but not 7 days post‐CCI

Having demonstrated the effectiveness of human Treg on both human and rat immune cells in vitro, we sought to determine whether Treg could modulate the central immune response to CCI in our rodent model. First, we elected to study the effect of Treg on microglia activation at 7 days post‐CCI, as we have previously shown that MSC alter microglia activation at this time point.^12^ Treg were infused via tail vein at a dose of 10 × 10^6^ cell/kg at 24 hours after CCI. There were noted no adverse effects of Treg infusion.

**FIGURE 4:**
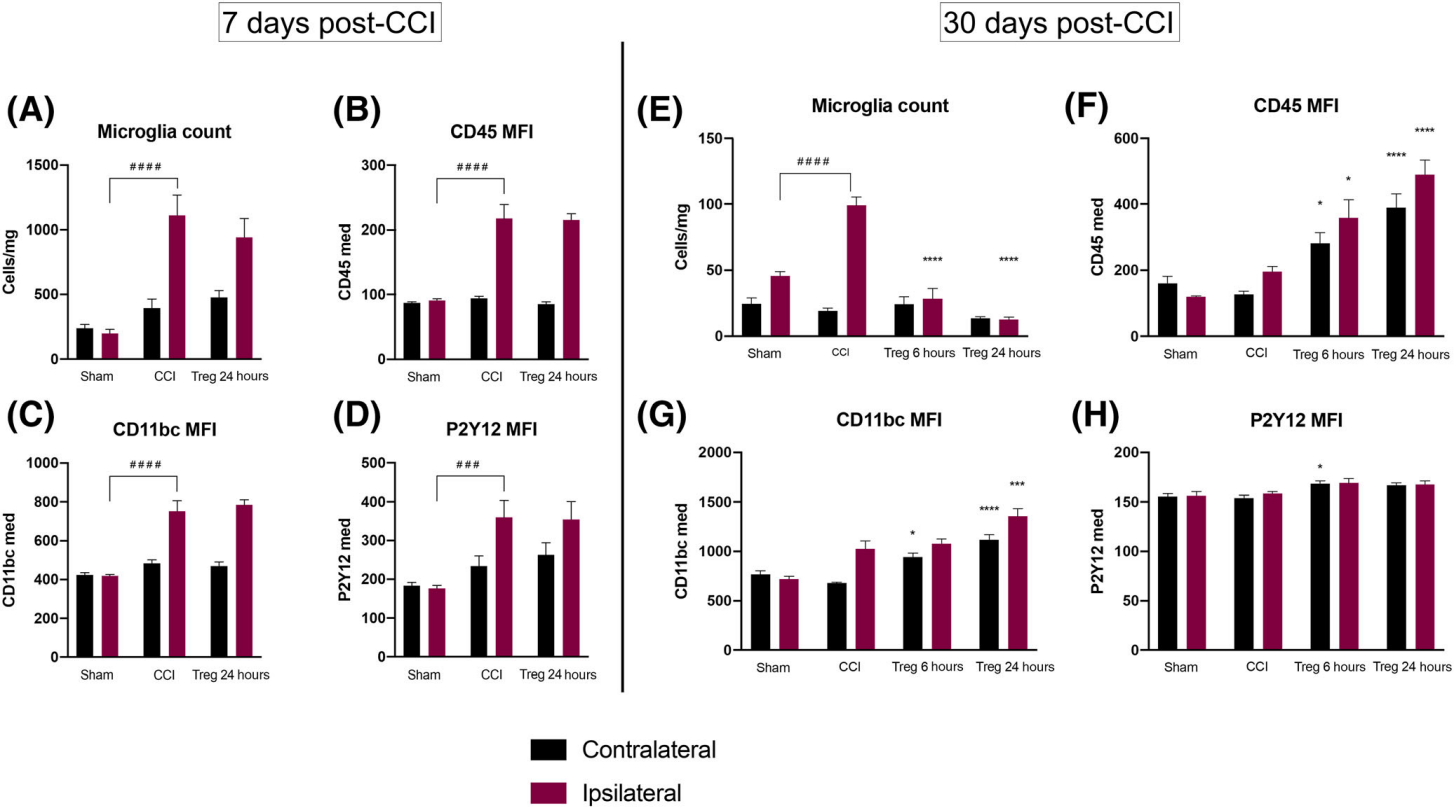
Flow cytometric characterization and comparison of microglia in the contralateral (uninjured) and ipsilateral (injured) hemispheres after CCI and Treg therapy at 7 days post‐CCI and 30 days post‐CCI. A, Absolute microglia counts, measured as cells/mg of brain tissue, at 7 days post‐CCI. B‐D, MFI of CD45, CD11bc, and P2Y12 expression 7 days post‐CCI, respectively. E, Absolute microglia counts, measured as cells/mg of brain tissue, at 30 days post‐CCI. F‐H, MFI of CD45, CD11bc, and P2Y12 expression 30 days post‐CCI, respectively. Statistical significance between sham and CCI is indicated with #*P* ≤ .05, ##*P* ≤ .01, ###*P* ≤ .001, and ####*P* ≤ .0001. Replicates: 7d cohort, N = 8; 30d cohort, N = 6. Statistical significance between CCI and Treg is indicated with *P ≤ .05, **P ≤ .01, ***P ≤ .001, and ****P ≤ 0.0001. CCI, controlled cortical impact; MFI, median fluorescent intensity; Treg, regulatory T cells

There were significant differences between sham and CCI in terms of absolute microglia counts and markers of activation at 7 days post‐CCI; however, there were no differences observed between CCI and Treg 24 hours. Specifically, there was a significant increase in the number of microglia cells in the ipsilateral (injured) hemisphere of the CCI group (Figure 4A). Furthermore, in the ipsilateral hemisphere of the CCI group, the microglia had higher levels of expression of the microglia markers CD45, CD11bc, and P2Y12 in comparison to sham (Figure 4B‐D). There were also significant changes in phenotypic markers after CCI, including increases in FSC, SSC, percentage and MFI of CD32, and percentage of RT1B (Figure S5). There was a significant decrease in RT1B MFI in the CCI group compared with sham (Figure S5).

In order to ensure that we infused Treg prior to initiation of the adaptive immune response, we elected to add an additional treatment time point at 6 hours post‐CCI when we examined the effects of Treg therapy at a chronic time point after CCI. At 30 days post‐CCI, we observed significant chronic microgliosis in the ipsilateral hemisphere of the CCI brain in comparison to sham (Figure 4E). However, there were no differences between CCI and sham with respect to the microglia markers CD45, CD11bc, and P2Y12 (Figure 4F‐H). The only differences in microglia phenotypic markers were increases in SSC and percentage of RT1B in the ipsilateral hemisphere and a decrease in RT1B MFI in the contralateral hemisphere of the CCI brain (Figure S5).

Treg therapy at both 6 and 24 hours significantly decreased the number of microglia present in the ipsilateral hemisphere after CCI (Figure 4E). There were no significant differences in microglia counts in the contralateral hemisphere between CCI and either treatment group at 30 days. Treg therapy led to significant increases intensity of the microglia identification markers CD45, CD11bc, and P2Y12. There were significant increases in expression CD45 and CD11bc MFI in both the Treg 6 hours group and Treg 24 hours groups compared with CCI (Figure 4F‐H). There was also a small, but significant, increase in P2Y12 expression in the contralateral hemisphere of the Treg 6 hours group compared with CCI. In addition, in the Treg 6 hours group, there was significant increase in CD32 MFI in the ipsilateral hemisphere compared with CCI (Figure S5). In the Treg 24 hours group, there was a bilateral increase in SSC (Figure S5).

Altogether, these data indicate that CCI leads to chronic microgliosis in the ipsilateral hemisphere that is attenuate by Treg therapy. Furthermore, Treg therapy led to significant changes in expression of microglia identification markers.

### 3.4 Treg infusion at 6 or 24 hours does not decrease BBB permeability after CCI

**FIGURE 5:**
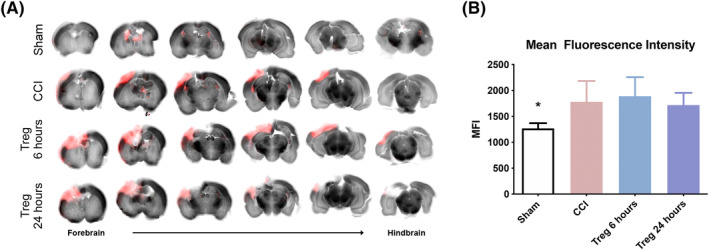
Assessment of BBB permeability at 96 hours after CCI. A, Representative slices of the brain, from forebrain to hindbrain, as imaged using a laser scanner system. B, Quantitative analysis of BBB permeability, as measured by MFI, demonstrates that there is significant difference between sham and CCI at 96 hours post‐CCI; however, neither Treg at 6 or 24 hours resulted in BBB improvement. N = 6. Statistical significance is indicated with *P ≤ .05, **P ≤ .01, ***P ≤ .001, and ****P ≤ .0001. BBB, blood‐brain barrier; CCI, controlled cortical impact; MFI, median fluorescent intensity; Treg, regulatory T cells

TBI leads to disruption of the BBB and long‐term increases in permeability, which can lead to worsened short‐ and long‐term outcomes. We have previously demonstrated that cellular therapy can restore vascular integrity to the BBB after CCI, therefore we evaluated the ability of Treg therapy to attenuate BBB permeability after CCI.^13^, ^20^ At 96 hours post‐CCI, we observed the expected significant difference in dye extravasation between the sham and CCI brains, indicating increased BBB permeability after CCI (Figure 5B). However, we did not observe any significant restoration of the BBB after Treg infusion at either 6 or 24 hours.

### 3.5 Treg infusion alters the endogenous rat lymphocyte populations in the spleen at both 96 hours and 30 days after CCI but alters blood lymphocyte only in the first 96 hours

**FIGURE 6:**
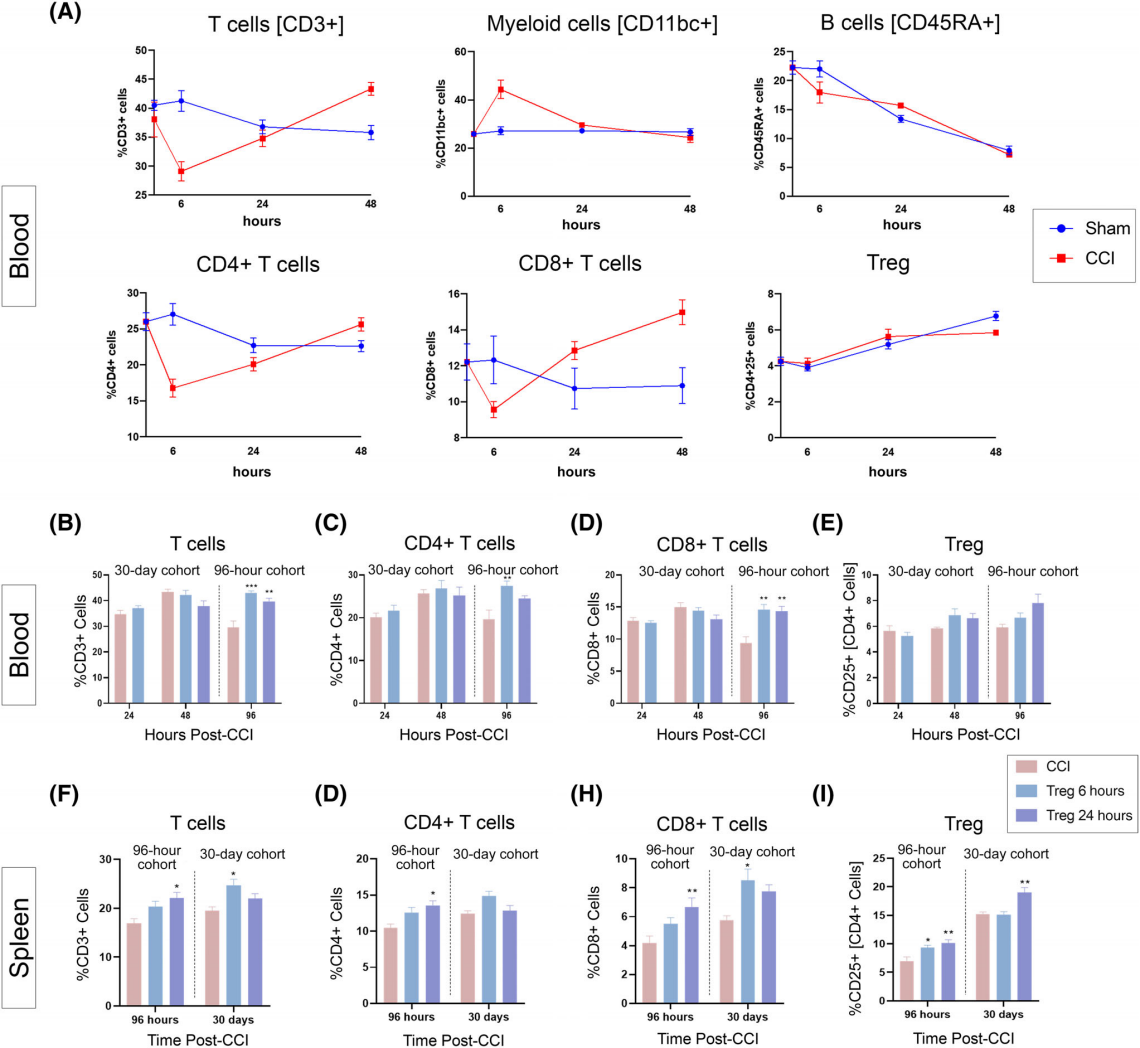
Flow cytometric characterization of immune cell populations in the blood and spleen after CCI and Treg therapy. A, Time course of changes in immune cell populations in the blood over the first 48 hours after CCI. B‐E, Effect of Treg therapy on T‐cell populations in the blood at 24, 48, and 96 hours after CCI. F‐I, Effect of Treg therapy on T‐cell populations in the spleen at 96 hours and 30 days after CCI. Replicates: 7d cohort, N = 8; 30d cohort, N = 6. Statistical significance is indicated with *P ≤ .05, **P ≤ .01, ***P ≤ .001, and ****P ≤ .0001. CCI, controlled cortical impact; Treg, regulatory T cells

In the 30‐day cohort, blood was collected at baseline, 6 hours post‐CCI, 24 hours post‐CCI, and 48 hours post‐CCI. Flow cytometry was then performed to identify rat lymphocyte and myeloid cell populations; Treg were identified as CD3+CD4+CD8‐CD25+ cells. There were several significant changes in cell populations after sham and CCI injuries (Figure 6A). However, there were no significant changes in cell populations in the blood after Treg infusion in the first 48 hours (Figure 6B‐E) or in the ratio of CD4+:CD8+ T cells (Figure S7).

In the 96‐hour cohort, however, there was a significant increase in the total CD3+ T‐cell and CD8+ T‐cell populations in both the Treg 6 hours group and Treg 24 hours group compared with CCI (Figure 6B,D). Furthermore, there was a significant increase in the CD4+ population in the Treg 6 hours group, but not the Treg 24 hours group, compared with CCI (Figure 6C). However, there were no significant differences in the ratio of CD4+:CD8+ T cells (Figure S7). In addition, there was a significant decrease in the percentage of B cells in both the Treg 6 hours group and Treg 24 hours group compared with CCI (Figure S6). There was no difference with respect to CD11bc myeloid cells.

With respect to splenocyte populations, at 96 hours post‐CCI, there was significant increase in the percentage of CD3+ T cells, as well as CD4+ and CD8+ T cells, in the Treg 24 hours compared with CCI (Figure 6F‐H). There was also a significant increase in the percentage of Treg within the CD4+ population in both the Treg 6 hours group and Treg 24 hours group compared with CCI (Figure 6I). In addition, Treg therapy had significant effects on the splenocyte populations at 30 days post‐CCI. There was significant increase in the total CD3+ T‐cell and CD8+ T‐cell populations in the Treg 6 hours compared with CCI (Figure 6F,H). In addition, there was a significant increase in the percentage of Treg within the CD4+ population in the Treg 24 hours group (Figure 6I). There were no differences in the percentage of CD4+ T cells (Figure 6G), B cells, or myeloid cells (Figure S6) in either Treg treatment group. Finally, there were no significant differences in the ratio of CD4+:CD8+ T cells at 96 hours or 30 days post‐CCI after treatment (Figure S7).

These data demonstrate the ability of Treg therapy to modulate the endogenous immune response after TBI that are significant at both subacute and chronic time points.

## 4 DISCUSSION

We have demonstrated that human UCB Treg effectively suppresses proinflammatory cytokine production in human and rat cells, including both rat microglia and splenocytes, in vitro. Furthermore, we have shown that Treg infusion augmented T‐cell populations in the blood and spleen and significantly inhibited microgliosis in the injured hemisphere at 30 days post‐CCI, indicating a potential to suppress the chronic secondary neuroimmune response within the brain that contributes to secondary brain injury and long‐term morbidity.^3^ However, Treg infusion did not affect changes in the microglia population at 7 days post‐CCI or improve BBB permeability at 96 hours post‐CCI, indicating that Treg may not directly interact with microglia or other CNS cells, such as astrocytes or cerebrovascular endothelial cells, in the acute time periods after injury.

Our in vitro data demonstrate the ability of Treg to effectively suppress proinflammatory adaptive immune responses by human PBMC and rat splenocytes. However, as the blood and spleen flow cytometry data suggest, Treg may only begin to modulate the endogenous adaptive immune system in the subacute time period after injury in vivo. In the first 48 hours post‐CCI, we did not observe any significant effects of Treg therapy on immune cell populations in the blood. At 96 hours post‐CCI, however, Treg infusion led to significant changes in the adaptive immune system in both the blood and spleen, specifically in increase in the percentage of T cells—including the CD4+, CD8+, and Treg subsets—and a decrease in the percentage of B cells.

TBI causes significant and chronic deficits in the adaptive immune system.^24^, ^25^, ^26^ Previous studies have demonstrated significant decreases in splenic T‐cell populations and increase in brain T‐cell populations at subacute and chronic time points after CCI in mice.^27^, ^28^ Our data demonstrate that Treg infusion led to chronic changes in the splenic T‐cell populations at 30 days post‐CCI, including increases in the percentage of CD8+ T cells and Treg. This ability to affect long‐lasting changes on the adaptive immune system may explain the ability of Treg to attenuate the chronic, but not subacute, microglia response after TBI. In a mouse CCI model, Krämer et al. demonstrated that depletion of Treg led to significant increase in T‐cell infiltration into the perilesional brain parenchyma; however, Treg depletion did not affect volume lesion size or microgliosis at 5 days postinjury.^29^ These results are consistent with our own in that exogenous administration of Treg did not affect BBB permeability or microgliosis at 7 days postinjury. However, we did not examine T‐cell infiltration into the brain at our subacute or chronic time points after Treg infusion.

In addition to chronic changes in the peripheral immune system, TBI causes significant and chronic alterations in microglia, the main immune effector cells in the CNS. The initial proinflammatory and phagocytic functions of microglia—removal of cell debris, clearance of foreign pathogens, and assistance in repair of the BBB—are likely beneficial, but to what extent and for what period of time is still under investigation.^3^ Prolonged microglia activation, which has been demonstrated in both rodents and humans after TBI, likely contributes to progressive morbidity and neurodegeneration.^3^, ^30^, ^31^, ^32^ Targeting the pathologic microglia population and augmenting the reparative, anti‐inflammatory functions of microglia could improve the efficacy of potential therapeutics. Our lab has previously demonstrated the cellular therapy, including MSC and MAPC improve BBB permeability, attenuate microglia activation and improve long‐term outcomes after TBI in preclinical models.^8^ Furthermore, previous work has shown that MAPC treatment increases the endogenous Treg populations in the spleen and blood, which was associated with in increased in neuroprotective microglia phenotypes after CCI.^9^

Treg infusion at both 6 and 24 hours post‐CCI significantly decreased the number of microglia present in the injured hemisphere at 30 days. With respect to our microglia phenotypic markers, Treg did not significantly alter P2Y12, our marker to distinguish microglia from peripheral macrophages, in the injured hemisphere; however, Treg did increase expression of both CD45 and CD11bc. Increased expression of CD45 has been associated with microglia activation and chronic peripheral inflammation in humans, while CD11b and CD11c regulate TLR‐4 signaling and recruitment of immune cells to sites of injury.^33^, ^34^, ^35^ We did not observe significant differences in the percentage CD32+ or RT1B+ microglia, markers of phagocytosis/immune cell clearance and antigen presentation, respectively.^36^, ^37^, ^38^, ^39^

Furthermore, our t‐SNE analyses also demonstrated that Treg infusion at 24 hours altered the microglia population at 30 days and decreased the number of cells present in the CCI‐specific clusters. Interestingly, these CCI‐specific cell clusters at 30 days post‐CCI had high expression of CD45 and CD11bc, as well as high FSC and SSC, suggesting that these could be activated microglia. This discrepancy between traditional flow cytometry analyses and t‐SNE, a machine learning visualization technique, deserves further study.

The question remains as to whether the microglia present at 30 days post‐CCI and after Treg infusion were functionally reparative, harmful, or homeostatic. We still lack a clear understanding of microglia phenotypes after TBI, especially at chronic time points, and how these phenotypes affect changes in neurodegeneration and morbidity. The paradigm that microglia exist as discrete subsets of the classical proinflammatory M1 and anti‐inflammatory M2 dichotomy has largely fallen out of favor.^40^ However, further investigation into differentiated functional phenotypes (eg, IL‐10 and transforming growth factor beta 1 [TGF‐β1] expression), metabolomic phenotypes and changes in gene expression of microglia after TBI and subsequent therapy, as demonstrated by recent studies, may provide more insight into this question.^41^, ^42^ Furthermore, as demonstrated by our in vitro and in vivo data, Treg likely inhibited chronic microgliosis via a combination of paracrine and direct effects on the systemic adaptive immune system, and potentially direct interaction with microglia. Further investigation into these mechanisms will help improve future efforts towards translation of Treg therapy for TBI.

## 5 CONCLUSIONS

Our data demonstrate that human Treg therapy altered both the peripheral and central immune responses after TBI in our rodent model and significantly attenuated chronic microgliosis in the injured hemisphere. Furthermore, our in vitro data show that Treg effectively inhibit proinflammatory immune responses in rat splenocyte and microglia. With recent improvements in isolation and expansion techniques, we now have the capability to produce sufficient number of Treg, from both autologous and nonautologous (ie, UCB) sources, for use in clinical trials. Treg therapy is a new potential cellular therapeutic to improve outcomes after TBI.

## 6 CONFLICT OF INTEREST

S.D.O. has received research support from Athersys, CBR Systems, Hope Bio, and Biostage. C.S.C. has received research support from Athersys, CBR Systems, Hope Bio, and Biostage, and is on the Scientific Advisory Board of Cellvation, Biostage, and CBR. The other authors indicated no potential conflict of interest.

## AUTHOR CONTRIBUTIONS

H.W.C., K.S.P., S.D.O.: conception and design, final approval of manuscript, collection and/or assembly of data, data analysis and interpretation; C.S.C.: conception and design, final approval of manuscript, data analysis and interpretation; N.E.T.F.: collection and/or assembly of data, data analysis and interpretation; A.K., C.M., L.C., N.F.M., A.S.B.: collection and/or assembly of data.

7

## Supporting information


**Figure S1**. Flow cytometric characterization of human UCB Treg populations immediately after isolation (left) and post‐thaw (right). Treg were identified as CD3‐positive, CD4‐positive, CD8‐negative, CD25‐positive, and CD127‐dim cells
**Figure S2**. Immunohistochemical staining of cultured rat microglia. IBA1 (left) was used to identify microglia; Hoescht (center) was used to identify nuclei. Overlay of IBA1 and Hoescht (right) demonstrate that nearly all cultured cells were IBA1+ microglia. A digital enlargement is provided to better visualize cell morphology. Scale bar: 100 μm
**Figure S3**. Flow cytometric gating strategy to identify microglia. A,B, The single cell population was identified based on SSC and FSC. C, Live cells were identified as negative for the Ghost viability dye. Microglia were identified using a two‐set method. First, P2Y12+ (BV421) cells, D, were identified. The P2Y12+ cells were then gated on CD11bc (PE‐Cy7) and CD45 (APC‐Cy7). Microglia were identified as triple‐positive cells. SSC, side scatter; FSC, forward scatter
**Figure S4**. Flow cytometric characterization of human UCB Treg on the rat peripheral immune cell panel. Human UCB Treg and rat blood were stained with the anti‐rat antibodies used in the rat immune cell panel. Comparison of CD4 and CD8 staining is shown here. The human UCB Treg (top left) were not positive for either CD4 or CD8, while the rat blood (top right, gated on CD3+ T cells) demonstrated positive staining for both markers. The same human UCB Treg had positive staining for the human CD4 antibody (bottom)
**Figure S5**. Additional flow cytometric characterization and comparison of microglia in the contralateral (uninjured) and ipsilateral (injured) hemispheres after CCI and Treg therapy at 7 days post‐CCI (left; A‐F) and 30 days post‐CCI (right; A‐F). Statistical significance between sham and CCI is indicated with (#) for *P* ≤ .05, (##) for *P* ≤ .01, (###) for *P* ≤ .001, and (####) for *P* ≤ .0001. Statistical significance between CCI and Treg 24 hours is indicated with *P ≤ 0.05, **P ≤ .01, ***P ≤ .001, and ****P ≤ .0001. CCI, controlled cortical impact; MFI, median fluorescent intensity
**Figure S6**. Flow cytometric characterization of myeloid (CD11bc+) and B‐cell (CD45RA+) populations in the blood and spleen after CCI and Treg therapy. A, Effect of Treg therapy on myeloid and B cell populations in the blood at 24, 48, and 96 hours after CCI. B, Effect of Treg therapy on myeloid and B cell populations in the spleen at 96 hours and 30 days after CCI. Statistical significance is indicated with *P ≤ .05, **P ≤ .01, ***P ≤ .001, and ****P ≤ .0001. CCI, controlled cortical impact
**Figure S7**. Flow cytometric characterization of the ratio of CD4+:CD8+ T cells in the blood and spleen after CCI and Treg therapy. Treg therapy did not significantly impact the ratio of CD4+:CD8+ T cells at any time point in the spleen or blood. Statistical significance is indicated with *P ≤ .05, **P ≤ .01, ***P ≤ .001, and ****P ≤ .0001. CCI, controlled cortical impact

## Data Availability

The data sets generated during and/or analyzed during the current study are available from the corresponding author on reasonable request.
