# Supplementary material for: Human cord blood‐derived regulatory T‐cell therapy modulates the central and peripheral immune response after traumatic brain injury
Source: Stem Cells Transl Med. 2020 May 7;9(8):903–16. doi: 10.1002/sctm.19-0444 (PMC7381810; doi:10.1002/sctm.19-0444)
Supplement: Supplementary file 1 — Figure S1. Flow cytometric characterization of human UCB Treg populations immediately after isolation (left) and post‐thaw (right). Treg were identified as CD3‐positive, CD4‐positive, CD8‐negative, CD25‐positive, and CD127‐dim cells Figure S2. Immunohistochemical staining of cultured rat microglia. IBA1 (left) was used to identify microglia; Hoescht (center) was used to identify nuclei. Overlay of IBA1 and Hoescht (right) demonstrate that nearly all cultured cells were IBA1+ microglia. A digital enlargement is provided to better visualize cell morphology. Scale bar: 100 μm Figure S3. Flow cytometric gating strategy to identify microglia. A,B, The single cell population was identified based on SSC and FSC. C, Live cells were identified as negative for the Ghost viability dye. Microglia were identified using a two‐set method. First, P2Y12+ (BV421) cells, D, were identified. The P2Y12+ cells were then gated on CD11bc (PE‐Cy7) and CD45 (APC‐Cy7). Microglia were identified as triple‐positive cells. SSC, side scatter; FSC, forward scatter Figure S4. Flow cytometric characterization of human UCB Treg on the rat peripheral immune cell panel. Human UCB Treg and rat blood were stained with the anti‐rat antibodies used in the rat immune cell panel. Comparison of CD4 and CD8 staining is shown here. The human UCB Treg (top left) were not positive for either CD4 or CD8, while the rat blood (top right, gated on CD3+ T cells) demonstrated positive staining for both markers. The same human UCB Treg had positive staining for the human CD4 antibody (bottom) Figure S5. Additional flow cytometric characterization and comparison of microglia in the contralateral (uninjured) and ipsilateral (injured) hemispheres after CCI and Treg therapy at 7 days post‐CCI (left; A‐F) and 30 days post‐CCI (right; A‐F). Statistical significance between sham and CCI is indicated with (#) for P ≤ .05, (##) for P ≤ .01, (###) for P ≤ .001, and (####) for P ≤ .0001. Statistical significance between CCI and Tr [file SCT3-9-903-s001.pdf]

Supplemental Figure 1. Flow cytometric characterization of human UCB Treg populations immediately after isolation (left) and post-thaw (right). Treg were identified as CD3 positive, CD4 positive, CD8 negative, CD25 positive, and CD127 dim cells.

Supplemental Figure 2. Immunohistochemical staining of cultured rat microglia. IBA1 (left) was used to identify microglia; Hoescht (center) was used to identify nuclei. Overlay of IBA1 and Hoescht (right) demonstrate that nearly all cultured cells were IBA1+ microglia. A digital enlargement is provided to better visualize cell morphology. Scale bar: 100µm.

Supplemental Figure 3. Flow cytometric gating strategy to identify microglia. (A-B): The single cell population was identified based on SSC and FSC. (C): Live cells were identified as negative for the Ghost viability dye. Microglia were identified using a two-set method. First, P2Y12+ (BV421) cells (D) were identified. The P2Y12+ cells were then gated on CD11bc (PE-Cy7) and CD45 (APC-Cy7). Microglia were identified as triple positive cells. SSC, side scatter; FSC, forward scatter.

Supplemental Figure 4. Flow cytometric characterization of human UCB Treg on the rat peripheral immune cell panel. Human UCB Treg and rat blood were stained with the anti-rat antibodies used in the rat immune cell panel. Comparison of CD4 and CD8 staining is shown here. The human UCB Treg (top left) were not positive for either CD4 or CD8, while the rat blood (top right, gated on CD3+ T cells) demonstrated positive staining for both markers. The same human UCB Treg had positive staining for the human CD4 antibody (bottom).

Supplemental Figure 5. Additional flow cytometric characterization and comparison of microglia in the contralateral (uninjured) and ipsilateral (injured) hemispheres after CCI and Treg therapy at 7 days post-CCI (Left; A-F) and 30 days post-CCI (Right; A-F). Statistical significance between sham and CCI is indicated with (#) for $p \leq 0.05$, (##) for $p \leq 0.01$, (###) for $p \leq 0.001$, and (####) for $p \leq 0.0001$. Statistical significance between CCI and Treg 24hr is indicated with (*) for $p \leq 0.05$, (**) for $p \leq 0.01$, (***) for $p \leq 0.001$, and (****) for $p \leq 0.0001$. CCI, controlled cortical impact; MFI, median fluorescent intensity.

Supplemental Figure 6. Flow cytometric characterization of myeloid (CD11bc+) and B cell (CD45RA+) populations in the blood and spleen after CCI and Treg therapy. **(A)**: Effect of Treg therapy on myeloid and B cell populations in the blood at 24, 48, and 96 hours after CCI. **(B)**: Effect of Treg therapy on myeloid and B cell populations in the spleen at 96 hours and 30 days after CCI. Statistical significance is indicated with (*) for $p \leq 0.05$, (**) for $p \leq 0.01$, (***) for $p \leq 0.001$, and (****) for $p \leq 0.0001$. CCI, controlled cortical impact.

Supplemental Figure 7. Flow cytometric characterization of the ratio of CD4+:CD8+ T cells in the blood and spleen after CCI and Treg therapy. Treg therapy did not significantly impact the ratio of CD4+:CD8+ T cells at any time point in the spleen or blood. Statistical significance is indicated with (*) for $p \leq 0.05$, (**) for $p \leq 0.01$, (***) for $p \leq 0.001$, and (****) for $p \leq 0.0001$. CCI, controlled cortical impact.

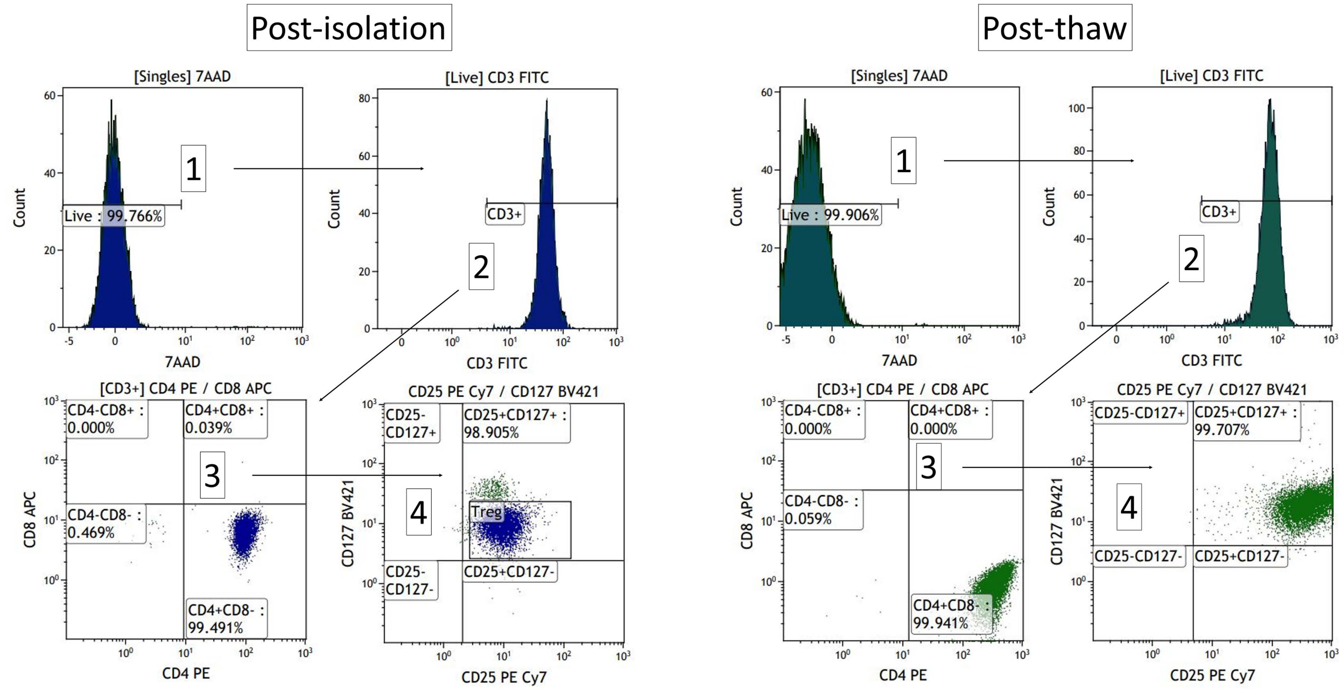

Post-isolation
Post-thaw
[Singles] 7AAD
Live : 99.766%
Count
7AAD
[Live] CD3 FITC
CD3+
CD3 FITC
[CD3+] CD4 PE / CD8 APC
CD4-CD8+ : 0.000%
CD4+CD8+ : 0.039%
CD4-CD8- : 0.469%
CD4+CD8- : 99.491%
CD8 APC
CD4 PE
CD25 PE Cy7 / CD127 BV421
CD25- CD127+
CD25+CD127+ : 98.905%
Treg
CD25- CD127-
CD25+CD127-
CD127 BV421
CD25 PE Cy7
[Singles] 7AAD
Live : 99.906%
[Live] CD3 FITC
CD3+
[CD3+] CD4 PE / CD8 APC
CD4-CD8+ : 0.000%
CD4+CD8+ : 0.000%
CD4-CD8- : 0.059%
CD4+CD8- : 99.941%
CD25 PE Cy7 / CD127 BV421
CD25-CD127+
CD25+CD127+ : 99.707%
CD25-CD127-
CD25+CD127-
1
2
3
4

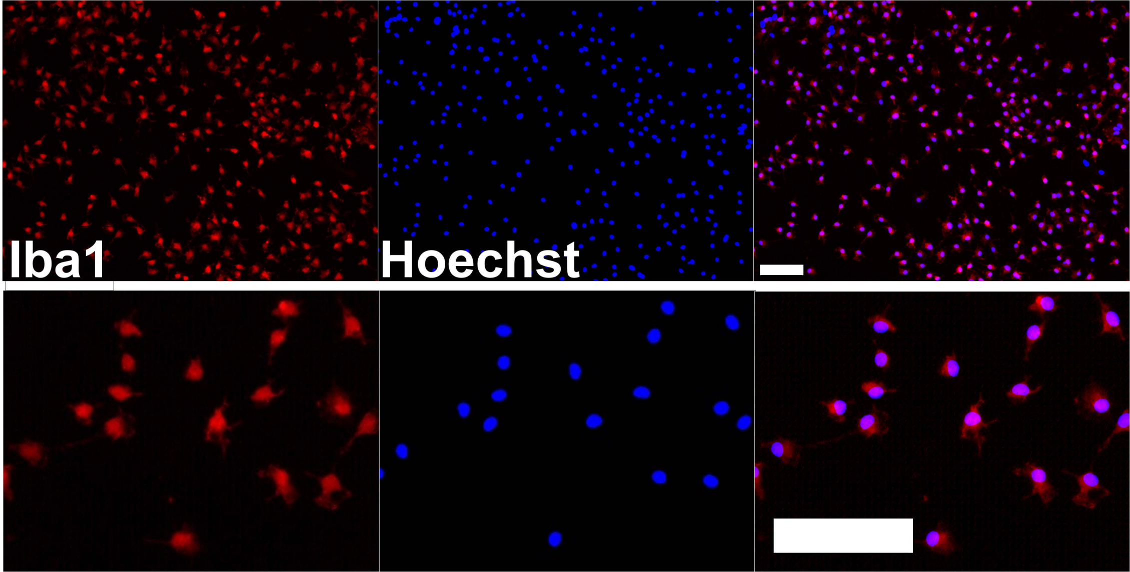
Iba1
Hoechst

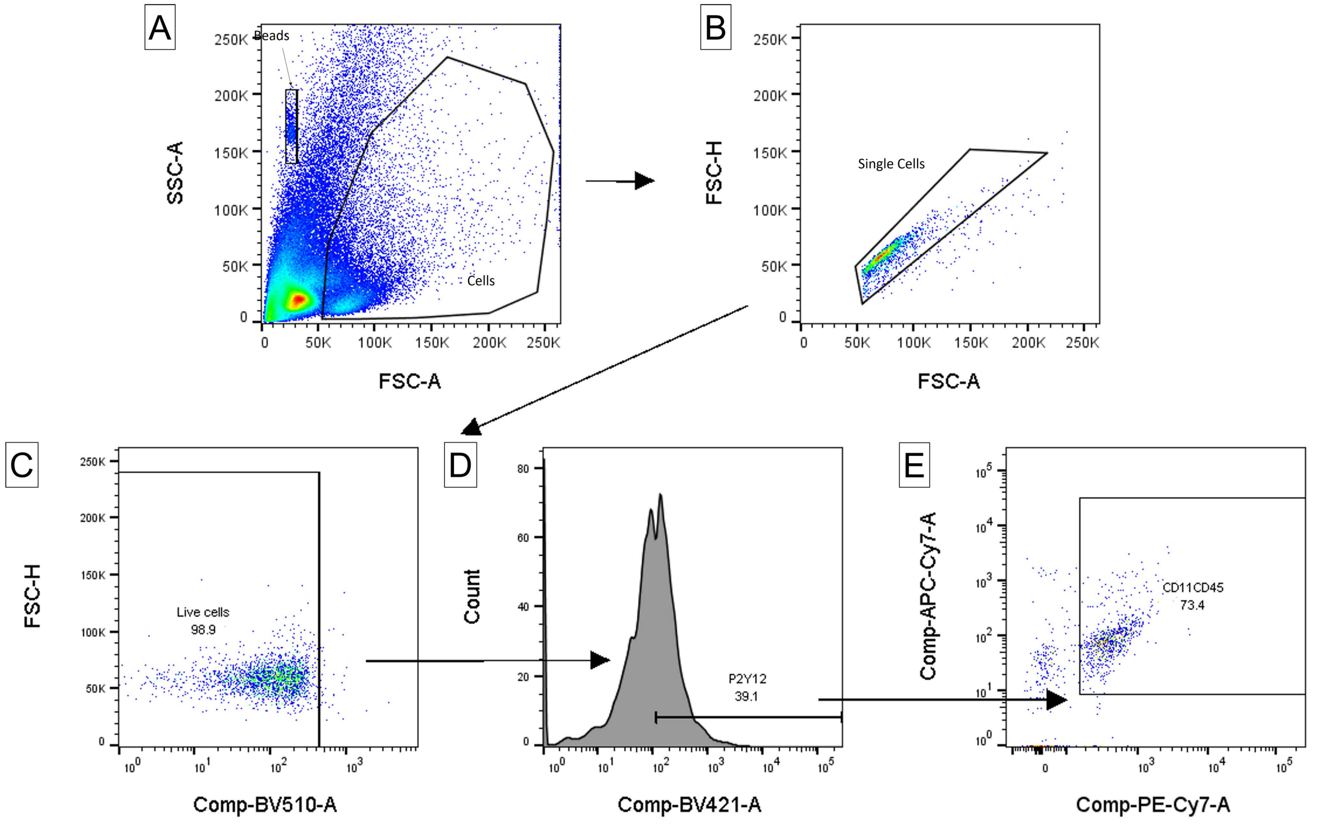
A
Beads
Cells
SSC-A
FSC-A
B
Single Cells
FSC-H
FSC-A
C
Live cells
98.9
FSC-H
Comp-BV510-A
D
P2Y12
39.1
Count
Comp-BV421-A
E
CD11CD45
73.4
Comp-APC-Cy7-A
Comp-PE-Cy7-A

Human Treg

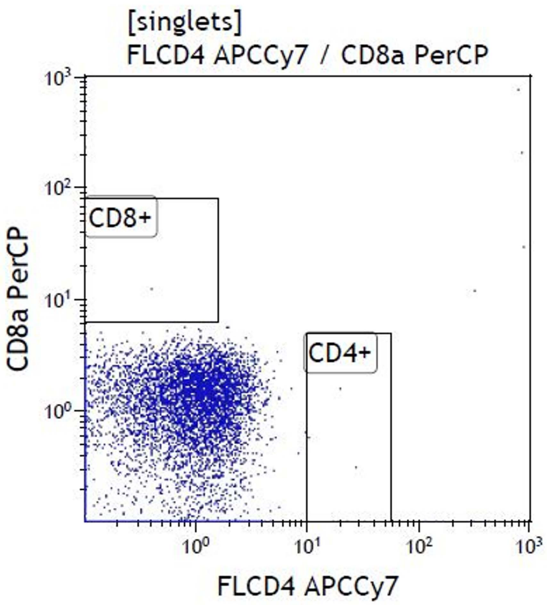

Rat Blood

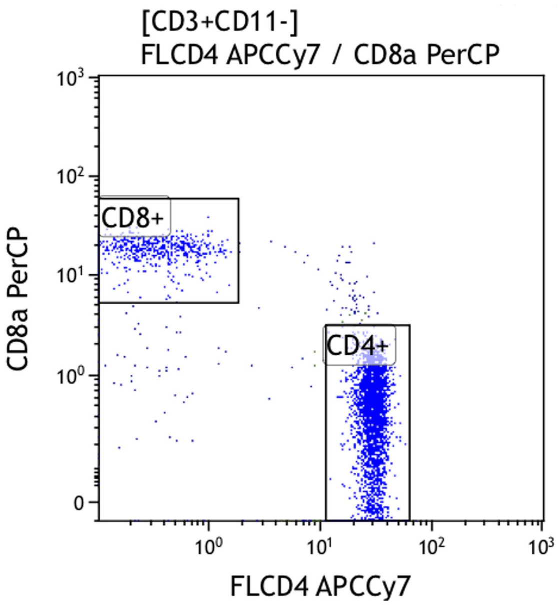

Human Treg

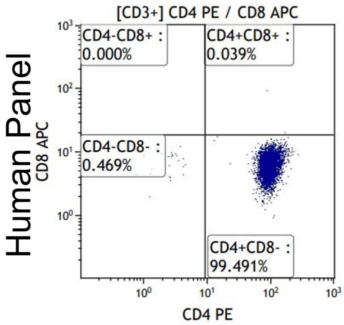

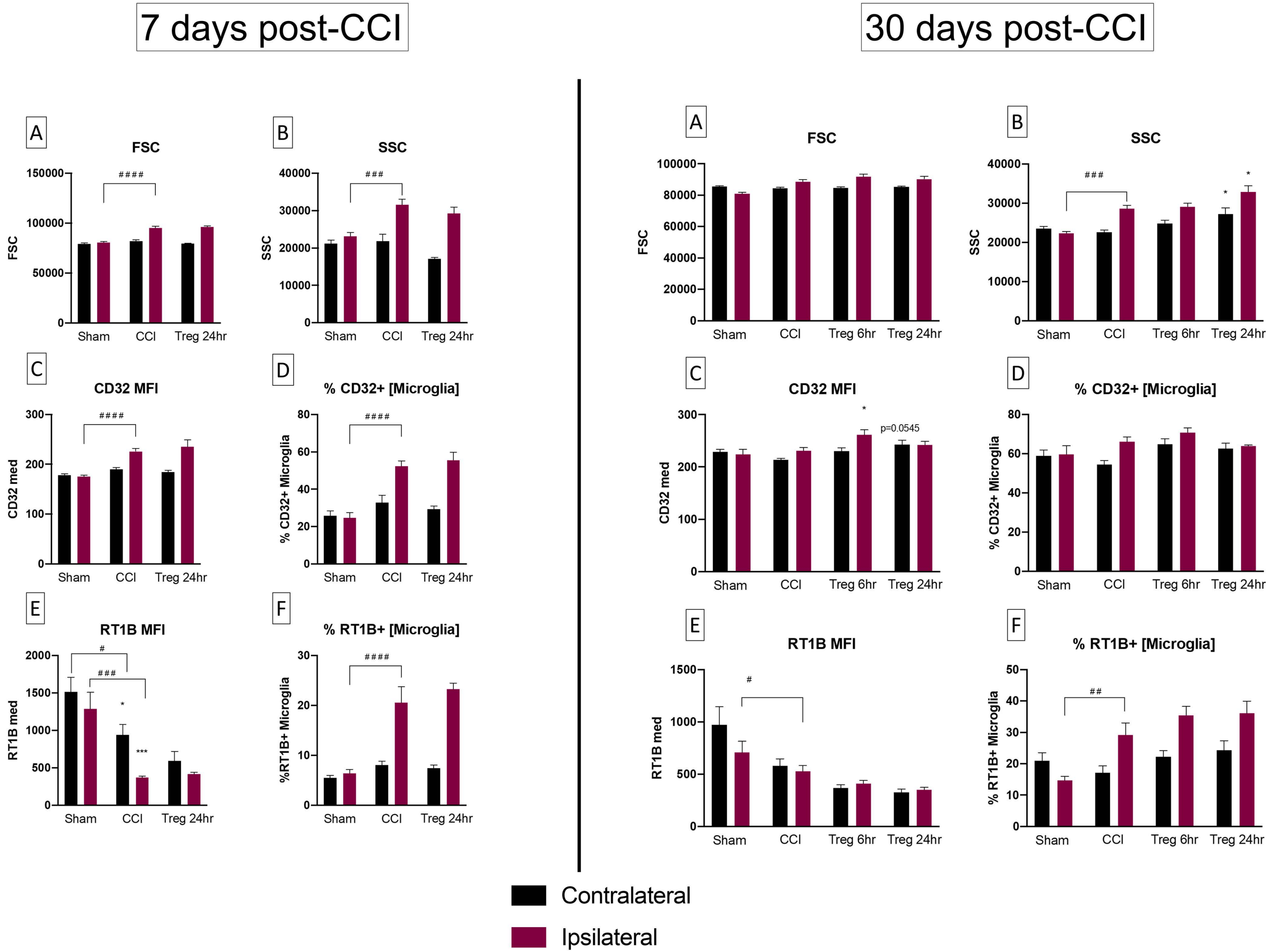
7 days post-CCI
30 days post-CCI
A
FSC
B
SSC
C
CD32 MFI
D
% CD32+ [Microglia]
E
RT1B MFI
F
% RT1B+ [Microglia]
CD32 med
% CD32+ Microglia
RT1B med
%RT1B+ Microglia
Sham
CCI
Treg 6hr
Treg 24hr
p=0.0545
Contralateral
Ipsilateral

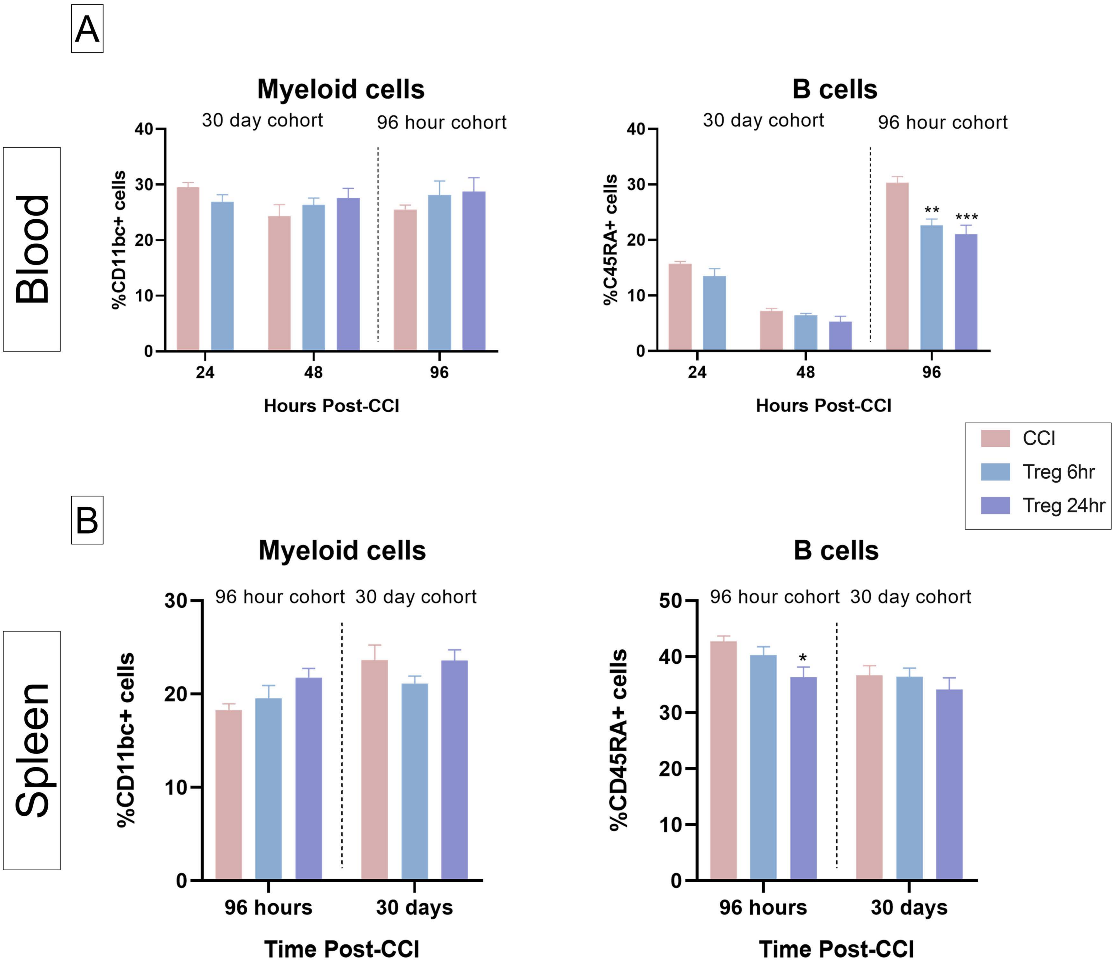
A
Blood
Myeloid cells
30 day cohort
96 hour cohort
%CD11bc+ cells
0
10
20
30
40
24
48
96
Hours Post-CCI
B cells
30 day cohort
96 hour cohort
%C45RA+ cells
**
***
0
10
20
30
40
24
48
96
Hours Post-CCI
CCI
Treg 6hr
Treg 24hr
B
Spleen
Myeloid cells
96 hour cohort
30 day cohort
%CD11bc+ cells
0
10
20
30
96 hours
30 days
Time Post-CCI
B cells
96 hour cohort
30 day cohort
%CD45RA+ cells
*
0
10
20
30
40
50
96 hours
30 days
Time Post-CCI

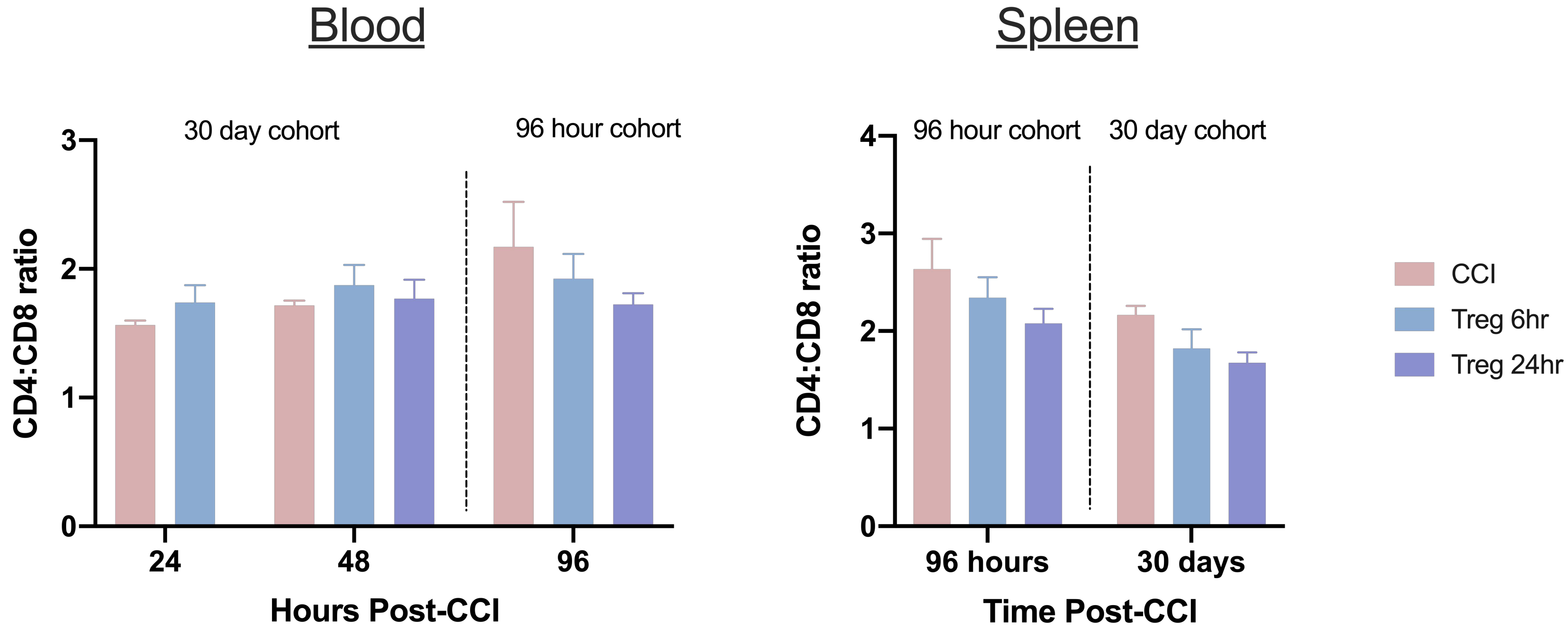
Blood
Spleen
30 day cohort
96 hour cohort
96 hour cohort
30 day cohort
CD4:CD8 ratio
0
1
2
3
4
24
48
96
Hours Post-CCI
96 hours
30 days
Time Post-CCI
CCI
Treg 6hr
Treg 24hr
